# Operation of Thin-Film Electrolyte Metal-Supported Solid Oxide Fuel Cells in Lightweight and Stationary Stacks: Material and Microstructural Aspects

**DOI:** 10.3390/ma9090762

**Published:** 2016-09-08

**Authors:** Daniel Roehrens, Ute Packbier, Qingping Fang, Ludger Blum, Doris Sebold, Martin Bram, Norbert Menzler

**Affiliations:** 1 Forschungszentrum Jülich GmbH, Institute of Energy and Climate Research, IEK-1: Materials Synthesis and Processing, Jülich 52425, Germany; d.sebold@fz-juelich.de (D.S.); m.bram@fz-juelich.de (M.B.); n.h.menzler@fz-juelich.de (N.M.); 2 Forschungszentrum Jülich GmbH, Institute of Energy and Climate Research, IEK-3: Electrochemical Process Engineering, Jülich 52425, Germany; u.packbier@fz-juelich.de (U.P.); q.fang@fz-juelich.de (Q.F.); l.blum@fz-juelich.de (L.B.); 3 Christian Doppler Laboratory for Interfaces in Metal-Supported Electrochemical Energy Converters, Jülich 52425, Germany

**Keywords:** metal-supported solid oxide fuel cell, thin-film electrolyte, stack operation, gas-flow sputtering, diffusion barrier layers

## Abstract

In this study we report on the development and operational data of a metal-supported solid oxide fuel cell with a thin film electrolyte under varying conditions. The metal-ceramic structure was developed for a mobile auxiliary power unit and offers power densities of 1 W/cm^2^ at 800 °C, as well as robustness under mechanical, thermal and chemical stresses. A dense and thin yttria-doped zirconia layer was applied to a nanoporous nickel/zirconia anode using a scalable adapted gas-flow sputter process, which allowed the homogeneous coating of areas up to 100 cm^2^. The cell performance is presented for single cells and for stack operation, both in lightweight and stationary stack designs. The results from short-term operation indicate that this cell technology may be a very suitable alternative for mobile applications.

## 1. Introduction

Materials science and engineering has been a major contributor to the progress of fuel cell technology [1]. Especially, solid oxide fuel cells (SOFCs) have attracted a great deal of interest because of their fuel flexibility, versatility, and efficiency [2,3]. The advancement of oxide ion electrolytes and the introduction of potent mixed ionic and electronic conducting (MIEC) electrodes has enabled a reduction in operating temperatures [4,5,6]. This made it possible to incorporate metallic interconnects into the cell design at substantially lower costs than for their ceramic counterparts [7,8]. Additionally, the development of materials and microstructures led to significant improvements in terms of power density and lifetime [9,10,11,12]. However, production costs for conventional ceramic SOFCs and limited mechanical robustness remain limiting factors.

Recent advances in powder-metallurgy have led to the establishment of another SOFC concept: the metal-supported SOFC (MSC) [13]. Here, the electrochemically active ceramic cell is constructed on top of a porous, and usually highly corrosion-resistant, steel support. MSCs have been demonstrated to be a promising technology for operation under non-stationary conditions because of their comparatively high tolerance of thermal, mechanical, and chemical stresses [14,15]. Additionally, the incorporation of standardized metal parts as a backbone permits relatively cheap mass manufacturing, which is crucial in terms of commercial competitiveness of the technology.

In recent years, many different concepts have emerged enabling cheaper manufacturing and assembly, as well as increased mechanical stability [14,16,17,18,19,20,21,22,23,24]. Of these, the Plansee SE’s cell design has gained considerable attention, since it is manufactured by a combined sintering and deposition route, leading to a very thin electrolyte membrane being realized [25,26,27]. These cells have been manufactured on a pilot scale and achieve power densities comparable to their more mature ceramic SOFC counterparts [28]. However, this type of metal-supported SOFC represents a much younger technology and previous electrochemical characterizations have been carried out mostly on a single-cell level or for symmetrical model samples. To our knowledge, results of electrical performance characterization and long-term testing in a stack environment has so far not been published. In this study, we summarize recent results with the Plansee SE MSC both in terms of electrical operation for single-cell arrangements, as well as for lightweight designs and in stationary stacks. Microstructural features before and after operation were explored with electron microscopy and energy dispersive X-ray spectroscopy.

## 2. Results and Discussion

### 2.1. Single-Cell Test

**Figure 1:**
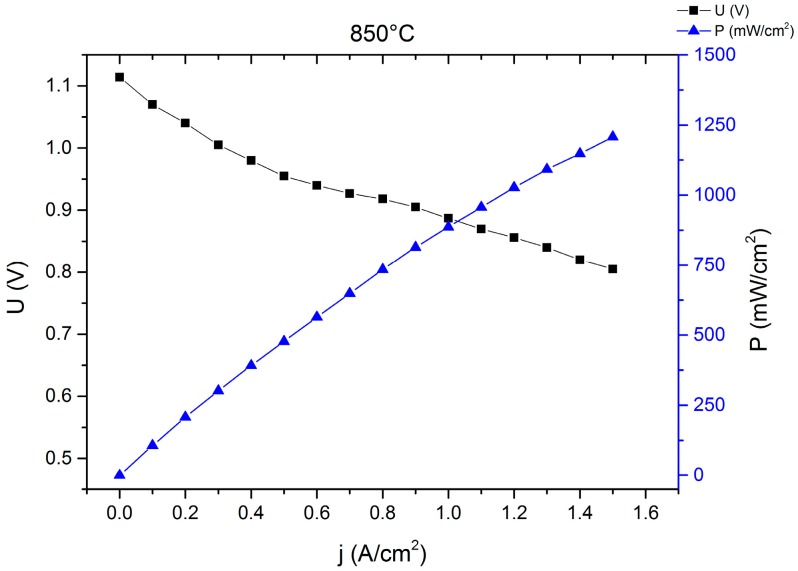
I–V curve of a planar 5 cm by 5 cm^2^ single cell MSC with a 4 µm thin layer 8YSZ electrolyte, a Ni/8YSZ anode and an LSCF cathode at 850 °C in 3% humidified hydrogen after 10 h of in-situ activation. For more details about the manufacturing refer to Section 3.

Single-cell MSCs with small-area cathodes (1 cm^2^) have been characterized under various conditions [26]. An example is given in Figure 1. This cell was activated in-situ for 10 h at 850 °C in a dual gas atmosphere (air/3% humidified hydrogen) in order to achieve densification and adhesion of the green La_0.58_Sr_0.4_Co_0.2_Fe_0.8_O_3-δ_ (LSCF) cathode to the barrier layer.

Although 10 h at 850 °C is not sufficient to ensure a sintering of the A-site-deficient LSCF cathode, the power densities obtained from single-cell tests with hydrogen are in a range that is comparable to full ceramic SOFCs (1.5 A/cm^2^ at 0.8 V and 850 °C). When the fuel gas is switched to a system-relevant simulated diesel reformate (50% N_2_, 15% H_2_ 14% CO, 11% H_2_O, 10% CO_2_) and a temperature of 750 °C cell performances in the range of 200 to 630 mW/cm^2^ at 0.7 V were recorded [26], which is sufficient for the application of this type of cell in a mobile APU.

### 2.2. Lightweight Cassette Stack

**Figure 2:**
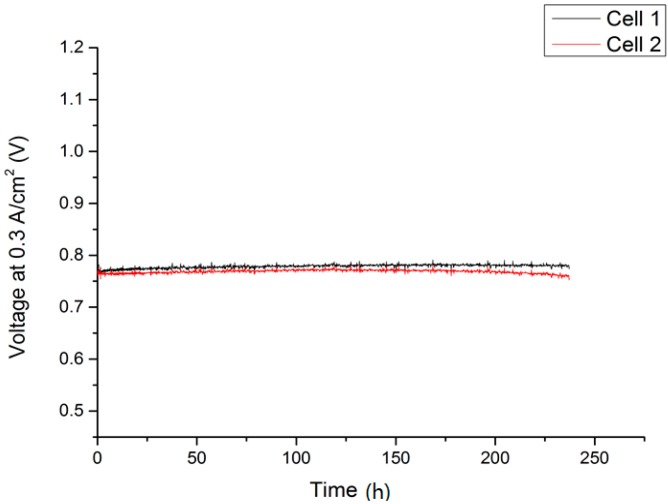
Performance of two metal-supported cells operated in a lightweight stack at 750 °C under galvanostatic conditions.

**Figure 3:**
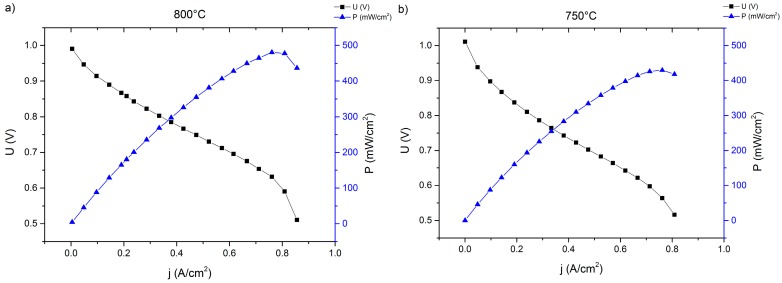
I-V- and I-P-curves of cell 1 of the lightweight MSC stack after joining, measured at 800 °C (**a**) and 750 °C (**b**).

A two-layer lightweight stack was set up with cells welded into the interconnector frame. After the glass sealant crystallized during the joining process for 100 h at 850 °C, gas tightness was achieved. The fuel gas was then set to humidified hydrogen and galvanostatic stack operation commencing at 750 °C, 0.3 A/cm^2^, and a fuel utilization of 20%. The resulting I–V and performance curves are shown in Figure 2 and Figure 3.

The measured IV-curves show a significantly reduced performance of the MSC (300 mA/cm^2^ at 0.8 V and 800 °C) as part of the stack compared to the single-cell measurements, both in terms of open circuit voltage (OCV) values and the slope of the voltage curve, which translates to a higher area-specific resistance. A lower OCV due to small internal leakages may be attributed to variations in the production cycle; the higher ASR compared to the single-cell test is a direct result of the introduction of several additional components which are necessary for stack operation, such as contacting oxide layers and or interconnector coatings. Due to the generally larger cell sizes, contact geometry and gas-flow are different compared to the single-cell laboratory experiment. Additionally, neighboring cells influence each other, for example with respect to temperature distribution [29]. These factors may contribute to the earlier onset of transport limitation at a current density of about 0.8 A/cm^2^, thus explaining the difference to the single cell measurement. However, these issues are common when comparing single-cell experiments with stack operation and a performance loss of 30%–50% is observed frequently [30].

**Figure 4:**
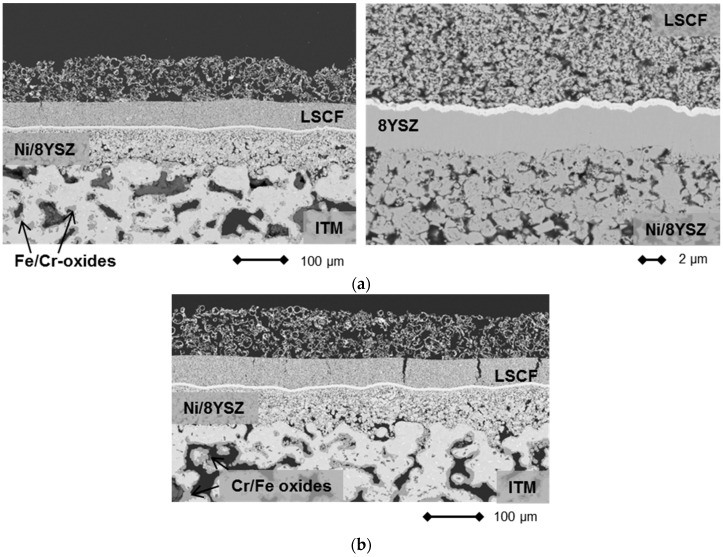
(**a**) SEM images of cross sections of cell 1 after operation at 750 °C under load (0.3 A/cm^2^). The dark gray areas within the metal substrate ITM (intermediate temperature metal, Plansee SE, Reutte, Austria) are comprised of chromium and iron oxides; and (**b**) SEM images of cross-sections of cell 2 after operation at 750 °C under load (0.3 A/cm^2^). The dark gray areas within the metal substrate ITM are comprised of chromium and iron oxides.

In terms of performance degradation over time, the data indicate different behavior comparing cell 1 and cell 2 in Figure 2. While the cell voltage for cell 1 increases by 5.2 mV over 237 h of operation, which is probably a result of a slight improvement of the activity at the cathode side, cell 2 shows a progressive degradation of 18.62 mV or 2.4% over 237 h, which equates to 10.2% over 1000 h. This was, however, not related to intrinsic defects on the cell level, since OCV values for both cells were similar at 1.011 V (cell 1) and 1.015 V (cell 2) at 750 °C after the joining process, but rather were a direct result of an external leakage and subsequent reoxidation of cell 2 that developed during the course of the experiment, which resulted in an area increase in dark gray oxidized domains and some cracks in the cathode layer in the scanning electron microscopy (SEM) analysis. Cell 1, on the other hand, was subjected to less microstructural degradation, as can be seen in Figure 4a. A cross-section of cell 2 is presented in Figure 4b.

Although the electrical performance of cell 1 did not show progressive degradation, an incipient corrosion of the metal substrate is visible as dark gray areas in Figure 4a (left). At the present time, it is unclear what the effect will be on the long term stability of the cell and the study of these issues is now part of research at the recently established Christian-Doppler Laboratory [31].

The Ni/8YSZ, however, was not subjected to severe microstructural changes and the thin film electrolyte was found to be crack-free for the entire surveyed area. Additionally, the LSCF-cathode activated in situ shows good adherence to the electrolyte membrane and the porosity distribution was found to be homogeneous.

### 2.3. Stationary Stack

**Figure 5:**
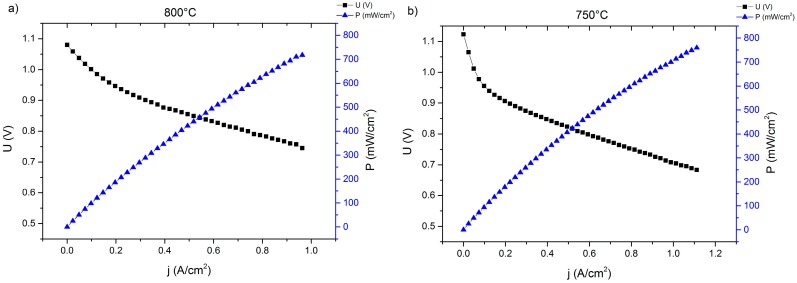
I-V- and I-P-curves of cell 1 during operation in a stationary stack after joining at 800 °C (**a**) and 750 °C (**b**).

In order to exclude influences characteristic of the lightweight stack design, an additional two-layer stack was set up according to a well-known stationary stack design. For this test, a higher, more system-relevant current density of 0.5 A/cm^2^ was selected. Before galvanostatic operation, an in situ activation of the cathode was conducted at 850 °C in dual gas conditions (air-side electrode: atmospheric air, fuel-side electrode: argon stream) for 100 h. After activation, IV curves were recorded with humidified hydrogen as fuel gas at different temperatures (see Figure 5).

Both I-V curves show a significantly higher performance of the Plansee MSCs compared to the corresponding results from the lightweight stack (Figure 3). This is true for both cells from both stacks and can be explained by the improved electrical contact in the stationary design. Additionally, both cells in the stationary design show a higher OCV-value because of improved internal and external gas-tightness and a later onset of the gas-transport limitation, which is a result of a more efficient layout of the distribution manifolds and gas channels compared to the lightweight system.

**Figure 6:**
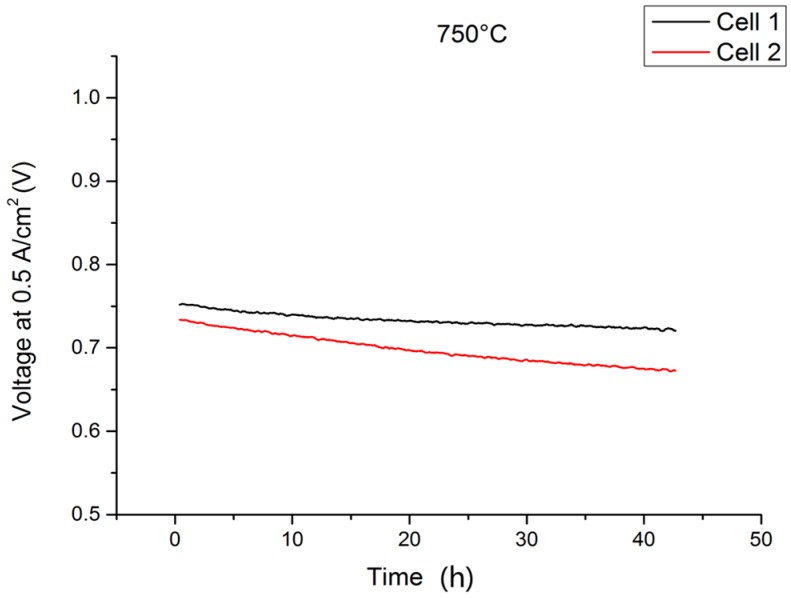
Performance of two MSC cells operated in a stationary stack at 750 °C under galvanostatic conditions (0.5 A/cm^2^).

**Figure 7:**
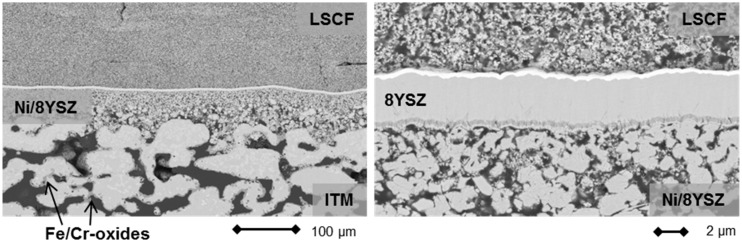
Cross-section of MSC 2 (see Figure 6) after operation in a stationary design.

The initial cell characteristics, as shown in Figure 5, were very promising. However, during the operation of the stack elevated degradation was recorded (see Figure 6). Over the course of galvanostatic operation (43 h), cell 1 lost 31 mV or 4.15% of the initial value, while cell 2 degraded by 61 mV (8.31%). Although the rate of performance loss in Figure 6 decreased considerably with increasing time at 0.5 A/cm^2^, we decided to cool down the stack and investigate the cells’ microstructure. Representative cross-sections are shown in Figure 7.

Although a slight internal leakage was detected after cooling down the stack, the thin 8YSZ electrolyte membrane was intact along the analyzed cross-sections (see Figure 7). No significant increase in local defect densities was observed compared to the initial state. Furthermore, the cathode microstructure and contact to the electrolyte were well established. EDS analysis of the cathode and electrolyte did not reveal any indications of a stoichiometric change in comparison to the as-prepared state.

**Figure 8:**
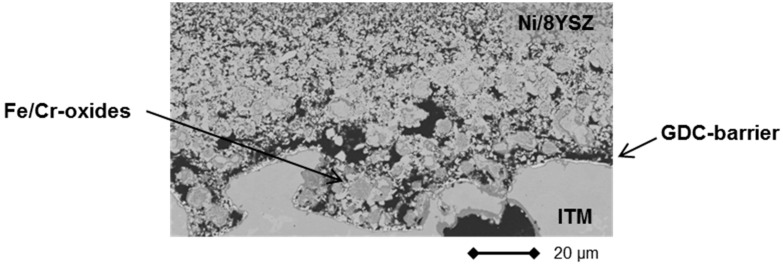
Cross-section of MSC 2 after operation in a stationary design exhibiting interdiffusion phenomena at contact interface between substrate/DBL/anode.

However, signs of progressive corrosion of the porous metal-support were found. The ITM substrate exhibited a large number of dark gray areas (see Figure 7, left), which were identified as Cr/Fe oxide phases by EDS analysis. These oxide areas are also found in direct contact with the Gd_2_O_3_-doped CeO_2_ (GDC) diffusion barrier, indicating insufficient inhibition of the cation transport under these conditions (see Figure 8).

These interdiffusion phenomena may have been caused by insufficient stability of the barrier material GDC under the applied conditions. Indeed, it has been reported previously that doped cerates are prone to oxygen loss and phase transformation under highly reducing atmospheres, which enables interaction with neighboring materials or even decomposition [32].

Higher performance degradation rates, compared to full ceramic SOFCs, are a relatively common phenomenon for MSCs and have been reported for various cell concepts by other groups [13,21,33,34,35]. Reasons for this are in part the presence of the porous steel support, which may oxidize or coarsen during operation and can, at least in part, interact with the catalytically active centers of the anode (usually Ni). By applying a suitable and more redox-stable barrier layer, the expected lifetime of the MSC will increase significantly, while electrical properties remain largely unaffected.

## 3. Materials and Methods

### 3.1. Cell Design and Manufacturing

**Figure 9:**
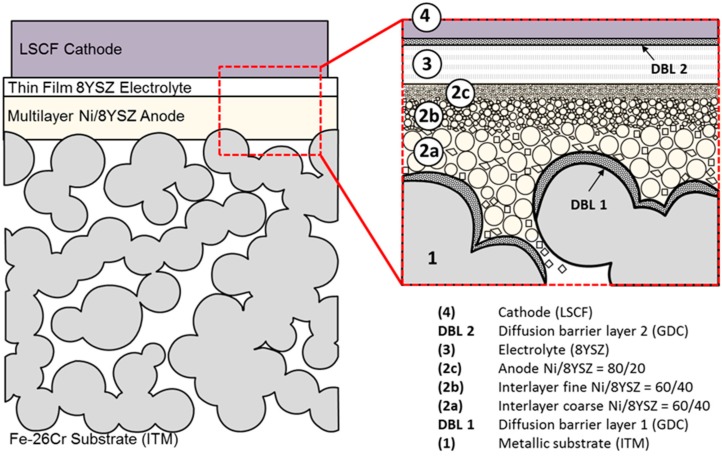
Schematic representation of the Plansee SE MSC. Ratios given for the composition of the anode in (2) are in weight percent.

The cell design is presented schematically in Figure 9. The first step in the production cycle is the manufacturing of the 0.8 mm thick porous metal support (Cr26-Fe, ITM) by a powder-metallurgical process. To avoid interdiffusion of Fe and Cr into the anode, the substrate is coated with a 500 nm thin Gd_2_O_3_-doped CeO_2_ (GDC) layer by magnetron sputtering. In the second step, a 40 µm multilayer nickel/8%-Y_2_O_3_-doped ZrO_2_ (Ni/8YSZ) anode is applied to the substrate by screen-printing and subsequently annealed in hydrogen atmosphere. The composition of the anode paste is varied in each step (finer particles, lower porosity) to ensure a graded improvement of the surface, which is necessary for the application of the 4 µm electrolyte membrane in step 3 via gas-flow sputtering (GFS). Finally, a 40 µm LSCF cathode is screen printed on top of a magnetron-sputtered 500 nm thin GDC diffusion barrier. Each step will be discussed in detail below.

#### 3.1.1. Substrate

**Figure 10:**
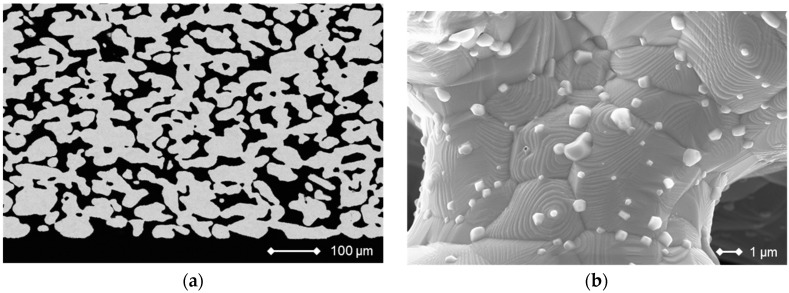
Cross section of the Cr26-Fe substrate material ITM (**a**) and fracture image (**b**). The bright crystals along the grain boundaries were identified as yttria and titania.

The substrate (ITM) consists of an oxide dispersion-strengthened (OSD) ferritic steel containing 26% chromium, which is alloyed with small amounts of titanium, molybdenum, and Y_2_O_3_ additions. The material was prepared by a powder-metallurgical process and exhibits porosities of about 40%, good corrosion resistance and a thermal expansion coefficient that matches SOFC materials well and was found to be independent of the porosity [36,37,38]. ITM was formed into sheets of 0.8 mm in thickness and different areas from 25 cm^2^ to 100 cm^2^, which can be welded to the interconnector frame. Figure 10 shows a cross-section and a fracture image, in which the oxide crystals along the grain boundaries are visible.

**Figure 11:**
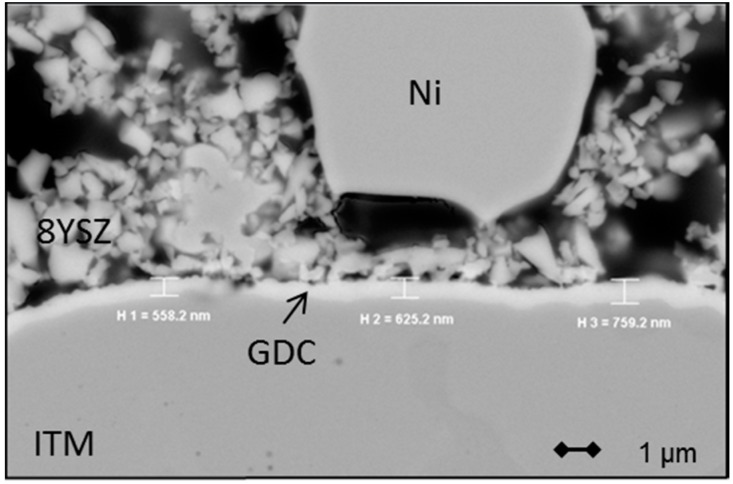
SEM image of a cross-section of an MSC with a magnetron sputtered GDC diffusion barrier layer covering the metal substrate.

The interdiffusion of cations from the metal substrate into the anode, and vice versa, can be a significant issue in the manufacturing and operation of MSCs and may lead to severe performance degradation [39]. Fe and Cr, once transported into the anode, form alloys with the Ni-catalyst and, thus, reduce cell performance. A number of possible protective coatings for different steel supports displaying sufficient chemical stability, a matching thermal expansion coefficient (TEC), and electrical conductivity have been explored in the literature and shown to be effective [39,40,41]. In this study we decided to produce a thin (0.5–1.0 µm) GDC layer by magnetron sputtering on top of the porous substrate, which is shown in Figure 11.

#### 3.1.2. Fuel-Side Electrode

In order to achieve a graded transfer from the large particle and pore sizes of the ITM substrate, a 3-step screen-printing process was developed. In contrast to the manufacturing of full ceramic SOFCs, which relies on atmospheric sintering of NiO, the screen printing pastes for the Plansee MSC are based on metallic Ni particles. To avoid high-temperature corrosion of the metal substrate, sintering has to be conducted in reducing atmosphere and anode porosities have to be defined by the organic content of the paste. Sintering is conducted at 1180 °C in hydrogen atmosphere.

**Figure 12:**
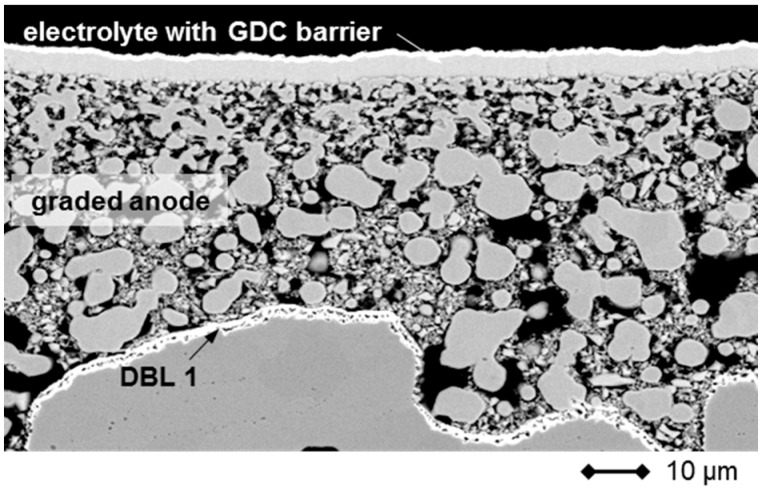
SEM-image of a cross section through the graded anode of an MSC before application of the cathode.

By gradually reducing the particle size of Ni in the screen-printing pastes and adjusting the solid load, composition and paste rheology a very smooth and homogeneous surface was achieved, which is necessary for the successful application of a thin 8YSZ electrolyte by gas-flow sputtering. A cross-section image of a sintered 3-layer anode is presented in Figure 12.

#### 3.1.3. Electrolyte Membrane

Dense and thin (4 µm) 8YSZ electrolytes have been prepared on top of the graded Ni/8YSZ anode by means of a gas-flow sputter process for active areas of up to 84 cm^2^. This method allows high deposition rates and a wide range of possible compounds and is generally considered to be a more economic physical vapor deposition (PVD) method [42].

**Figure 13:**
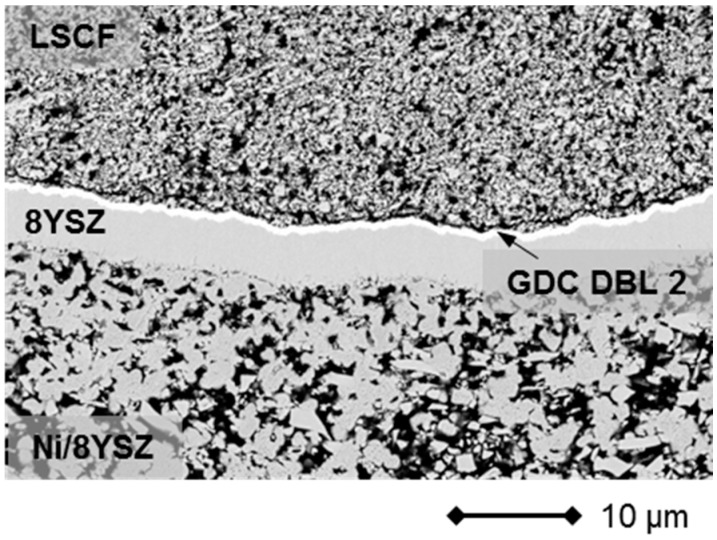
SEM image of a cross-section through an MSC highlighting the GFS applied 8YSZ electrolyte and the GDC diffusion barrier after operation. The cell was activated in situ for 100 h in a dual gas atmosphere at 850 °C and operated for 100 h at 750 °C.

After GFS processing, the 8YSZ membrane shows a low defect density and leakage rates of less than 3 × 10^−4^ hPa·dm^3^·s^−1^·cm^−2^ (Δp: 100 hPa) in air at room temperature have been obtained, which is sufficient for the electrical operation of the cell [27]. The 8YSZ layer adheres well to the anode surface and is able to withstand small height variations of the support even after operation (see Figure 13). Additionally, it was found that the thin membrane is flexible with respect to mechanical stresses which may arise from the oxidation of the anode or variations in thermal expansion coefficients [43]. This is a phenomenon which is characteristic of thin membranes, in general, and was recently reported for full ceramic SOFCs [44]. Due to the reactivity of the LSCF cathode material with zirconia, a dense 500 nm GDC layer is deposited by magnetron sputtering on top of the electrolyte in a second step to avoid the formation of strontium zirconates, which are detrimental to cell performance [45].

#### 3.1.4. Air-Side Electrode

Cathodes 40 µm in thickness were applied by screen printing on top of the electrolyte/diffusion barrier layer. Sr- and Fe-doped lanthanum cobaltites, in general, and A-site-deficient LSCF, in particular, have been used for SOFCs of all kinds and lead to excellent cell performance [46,47]. However, due to the presence of the metal substrate, a conventional sintering route at temperatures higher than 1000 °C in air is not possible for the MSC. Due to this, the cathode was kept in its green state during stack assembly and the first heat-up of the cell. An in situ activation step (850 °C, 10 to 100 h) in dual gas conditions was applied in order to ensure sufficient adherence to the electrolyte and, thus, good cell performance. The microstructure of the cathode after this activation step is shown in Figure 13 in Section 3.1.3.

#### 3.1.5. Stack Assembly

Two MSCs with an active area of 84 cm^2^ each were assembled into a lightweight cassette design that was developed for a mobile APU. The setup has been published earlier [26] and will only be discussed briefly. The interconnector frame was made up of dense ITM and accommodates the gas distribution manifolds. High temperature sealing was accomplished by using a conventional glass-ceramic [48]. During the joining process, the two-layer short stack was heated to 850 °C for 100 h in a dual gas atmosphere under a mechanical load of 0.5 kN. After that, the temperature was set to 750 °C and stack operation commenced under constant current conditions (0.3 A/cm^2^).

In addition to the lightweight setup, 10 × 10 cm^2^ MSCs were incorporated into a stack that was developed for stationary applications. Details can be found in a previous publication [29]. The joining process was identical to the lightweight-stack. Electrical operation was conducted galvanostatically at a lower temperature (700 °C) and under higher current density (0.5 A/cm^2^), compared to the lightweight setup, in order to expose the MSCs to conditions that were more relevant to the system’s point of operation.

For both stacks, humidified hydrogen was supplied to the fuel-side electrode (cassette stack Uf = 20%, stationary-stack Uf = 40%).

#### 3.1.6. Electron Microscopy

The SEM (scanning electron microscopy) images of polished cross sections were taken using a Zeiss Ultra55 (Oberkochen, Germany) with INCA Energy 355 EDX (energy-dispersive X-ray) and INCA Crystal EBSD (electron backscatter diffraction) detectors.

## 4. Conclusions

A metal-supported SOFC based on a porous steel substrate, a graded three-layer Ni/8YSZ anode, and a thin film 8YSZ membrane was operated in two different stack designs. The cells were characterized in terms of electrical performance and microstructural behavior. Single cell IV-characterization of such cells with an LSCF cathode activated in situ displays power densities of about 1 W/cm^2^ at 800 °C and 1.5 A/cm^2^. A two-layer lightweight MSC stack with an 84 cm^2^ active cell area was operated at 0.3 A/cm^2^ for more than 237 h at 750 °C. IV characterization of the cells activated in situ showed a reduction of the power densities obtained due to contacting and gas-transport losses, which is in line with other studies. A further two layer MSC stack in a stationary design was set up and operated at 0.5 A/cm^2^ for 43 h. For both stacks, one cell each showed increased degradation rates, while the other one remained relatively stable. The increased degradation rate is at least in part the result of setup related issues, which was confirmed by post-test microstructural analysis of the cells. Even though cell degradation for this MSC concept was higher compared to traditional full ceramic SOFCs, the first results from stack operation show performance data that are sufficient for the operation of a mobile APU, while a future redesign of current diffusion barrier layers will contribute greatly to extending cell lifetime.
